# Sex of household head and other household determinants of childhood anaemia among households in Ghana: regression analysis of the 2019 Malaria Indicator Survey

**DOI:** 10.1186/s41043-022-00327-5

**Published:** 2022-10-10

**Authors:** Desmond Klu, Percival Delali Agordoh

**Affiliations:** 1 Centre for Malaria Research, Institute of Health Research, University of Health and Allied Sciences, Ho, Ghana; 2 Department of Nutrition and Dietetics, School of Allied Health Sciences, University of Health and Allied Sciences, Ho, Ghana

**Keywords:** Childhood anaemia, Male-headed household, Female-headed household, Household factors, Children under age five, Ghana

## Abstract

**Background:**

Childhood anaemia is still a major public health concern. Although the prevalence of anaemia among children under age five is reducing in Ghana, the severity level is still worsening. This study sought to examine and compare household factors affecting the anaemia status of children under age five living in male- and female-headed households in Ghana**.**

**Methods:**

The study used a weighted sample of 5,799 household heads from the 2019 Ghana Malaria Indicator Survey (GMIS). A binary logistic regression analysis was used to examine the effect of sex of household heads and other household factors on the anaemia status of children under the age of five in male- and female-headed households in Ghana. All analyses were conducted at the 95% confidence level.

**Results:**

The results showed that a higher proportion (83.0%) of children under age five are not anaemic in households in Ghana. However, the probability of a child being anaemic is higher in male-headed households (aOR = 1.28; C.I:1.08–1.51), in the poorest (aOR = 2.41; CI: 1.59–3.65), poorer (aOR = 2.04; C.I:1.41–2.94) and middle (aOR = 1.78; C.I:1.29–2.46) household wealth category. Higher likelihood of anaemia was found among children in households that used charcoal (aOR = 1.51; C.I:1.15–1.99) and fuelwood (aOR = 1.44; C.I:1.02–2.02) for cooking. Similarly, there is a high probability of childhood anaemia in households with 5–10 members (aOR = 4.49; C.I: 3.78–5.34), 11 or more members (aOR = 7.21; C.I: 4.60–11.31) and household residing in northern part of Ghana (aOR = 1.40; C.I:1.07–1.83). The lower odds of being anaemic were recorded among children whose household heads were aged 40 years and older, household using other cooking fuels (aOR = 0.49; C.I: 0.21–0.78) and household with no bednets (aOR = 0.57; C.I: 0.44–0.74).

**Conclusions:**

The GMIS data suggest that anaemia and its severity are higher among children living in MHH than among those living in FHH. The results indicate that poverty, a higher number of household members, relatively younger male household heads and the type of cooking fuel used were factors accounting for the differences in childhood anaemia in MHH and FHH. Equal attention should be given to MHH and FHH in terms of programmes and interventions aimed towards preventing and reducing childhood anaemia in Ghana.

## Introduction

Anaemia has been defined as a condition in which the level of haemoglobin (Hb) in the human body is lower than normal, resulting in a decrease in the ability of red blood cells to transmit oxygen to body tissues [1, 2]. Of all population groups, the most vulnerable to anaemia are children. The World Health Organization (WHO) estimated that 60.2% of children were anaemic globally in 2019 [3].

According to WHO guidelines, children under 5 years of age with a haemoglobin concentration < 110 g/L are classified as anaemic [2]. The severity of anaemia is of three levels: mild (10.0–10.9 g/dL), moderate (7.0–9.9 g/dL) and severe (less than 7.0 g/dL) [3]. Iron deficiency is seen as the underlying cause of anaemia; infections that are acute and chronic infections resulting in inflammation and loss of blood, deficiencies in folate, vitamin B12 and vitamin A and hereditary traits inherited, such as thalassaemia, are considered other causes [4–6]. Other conditions, such as malaria, genetic disorders and cancer, are also exacerbated in anaemia in children [2, 5].

The condition of anaemia is often related to a high risk of maternal and child death, especially in Africa. For instance, childhood anaemia is estimated to have accounted for 5–18% of child deaths in Africa [6]. Furthermore, an estimated 3.3% of children under age five are severely anaemic in Africa, which is twice the global prevalence rate [3]. Anaemia due to iron deficiency has negative effects on the physical and mental development of children [7–9] and consequently manifests in indications such as heart failure, weakness, fatigue, shortness of breath and dizziness [10].

In Ghana, childhood anaemia prevalence has declined over the years. For example, anaemia among children less than five years of age decreased from 75% in 2003 to 66% in 2014 [11]. More recently, it has also decreased from 52.7% in 2016 to 42.5% in 2019 [12]. Malaria-related interventions have also been associated with a 60% decrease in the risk of anaemia among children [13]. Despite the reduction in childhood anaemia, anaemia levels (severity) increased from 1.9% in 2016 to 2.6% in 2019 [14]. This phenomenon raises the question of effective management of anaemia among children in Ghana.

The relationship between the household leader and the health status of household members has been examined by earlier studies [15–21]. The gender and sex dynamics of household headship, management and child health status have been profound in developing countries, including Ghana. Some studies have found that women in female-headed households (FHHs) are economically empowered and use resources to improve the health status, nutrition and well-being of children [22, 23]. Other studies strongly associate poverty and lack of resources with female household heads, hence concluding that children living in FHH have poorer health status than children in male-headed households (MHH) [16, 17, 24]. Furthermore, there is an increasing phenomenon of FHH in the African region, which is why it is necessary to demonstrate the effect of the household anaemia status of children [18]. However, only a few studies have studied gender dynamics in household headship and childhood anaemia [16, 25, 26].

This paper examined the effect of sex of household head and other household factors on anaemia status among children aged 6–59 households in Ghana using nationally representative data from the 2019 Malaria Indicator Survey.

## Methods

### Data source

Data for this study were obtained from the 2019 Ghana Malaria Indicator Survey (GMIS), which was conducted from September 25 to November 24, 2019. Data from the household file were utilized. The GMIS collects information on malaria prevention (ownership and use of treated mosquito bednets and assesses coverage of intermittent preventive treatment to protect pregnant women against malaria), anaemia levels in pregnant women and children, malaria treatment and prevalence in Ghana. In this study, data on a weighted sample household heads during the survey were extracted and analysed.

### Study setting

Ghana is a West African country that shares boundaries with Burkina Faso to the North, the Gulf of Guinea to the South, Togo to the East and Cote d'Ivoire to the West. It has a population of 30.8 million as of 2021 and has 16 administrative regions [27]. Accra is the capital of Ghana. Over the years, numerous interventions have been implemented in Ghana to combat anaemia, such as iron supplementation, food fortification, public education and sensitization, deworming and prevention and management of parasitic infections among children aged 6–59 months [28, 29].

### Survey and study participants

Details concerning the scope and methodology of the GMIS have already been published [12]. Briefly, the GMIS is a nationally representative survey conducted by the Ghana Statistical Service (GSS), the Ministry of Health (MOH) and the National Malaria Control Programme of the Ghana Health Service with technical support from the Inner City Fund (ICF) through the Demographic and Health Surveys (DHSs) Programme. The household questionnaire was used to list all usual household members and visitors of the 200 selected enumeration areas visited, and information was recorded on structures. In addition, information on the names of household heads and the global positioning system (GPS) coordinates of clusters were collected. A fixed number of 30 households from each group were selected to make up a total sample size of 6000 households that involved interviewing heads of household and all eligible women, taking data on age, sex of household heads, household source of water, toilet facilities, ownerships of various assets, ownership and use of mosquito nets. The questionnaire was used to identify women eligible for individual interviews and children aged 6–59 months eligible for anaemia and malaria testing with parental or guardian’s permission and consent.

### Sampling and sample size

The total number of weighted household heads (both male and female) in the GMIS 2019 was 5,799 comprising 3,782 male household heads and 2,017 female household heads.

### Study variables

#### Outcome variable

The outcome variable for this study was the anaemia status of children aged 6–59 months. Anaemia is defined in this study as a reduced level of haemoglobin in the blood, decreases the amount of oxygen reaching the tissues and organs of the body, and reduces their capacity to function. The initial categorization of the anaemia status of children aged 6–59 months was severe, moderate, mild anaemia and not anaemia. This was recategorized into two; thus, children under 5 years of age with severe, moderate and mild symptoms were coded “1” as being anaemic and “0” as not being anaemic.

Regarding the determination of the anaemia level among children under five years of age, a single-use retractable, spring-loaded, sterile lancet was used for finger or heel pricks. A drop of blood from the site was then collected in a microcuvette. The haemoglobin analysis was then conducted on site with a battery-operated portable HemoCue 201 + analyser, which produces a result in less than one minute. Anaemia test results were recorded both in the Biomarker Questionnaire and on a brochure that was left with the household members that also contained information on the causes and prevention of anaemia. Parents or guardians of children with haemoglobin levels below 8 g/dl (severe anaemia) were advised to go to a health facility and a referral letter with the haemoglobin reading to show to the health worker in the facility. Informed consent was sought from the respondents before collecting blood samples for testing for anaemia.

### Predictor variables

#### Main predictor variable

The main predictor variable used was sex of household headship. In the survey, this variable was categorized into two, namely (i) male household head and (ii) female household head

#### Other household factors

The following household factors were considered in the study: age of the household head (20–29, 30–39, 40–49, 50–59, 60–69, 70 +) and household wealth quintile (poorest, poorer, middle, richer, richest). The study also considered the size of household members (1–4 members, 5–10 members, 11 or more members), the number of treated bednets owned by the household (no bednets, 1–3 bednets, 4 or more bednets), the ecological zone of residence of the household (coastal/southern zone, middle belt, northern zone), the place of residence (urban, rural) and house/dwelling sprayed against malaria in the past 12 months (not sprayed, sprayed).

The other variables included the source of drinking water, type of toilet facility and the type of cooking fuel used by the household. The measurement and classification of the variable “*household source of drinking water” and the type of toilet facility used* were guided by the WHO/United Nations International Children’s Emergency Fund Joint Monitoring Programme for Water Supply, Sanitation and Hygiene (WHO/UNICEF-JMP) classification of the source of drinking water. For this study, the variable was classified into two categories: improved and unimproved sources of drinking water. In this study, the improved source of drinking water consisted of pipe borne, water within the dwelling, piped into the dwelling, pipe to yard/plot, piped to the neighbour’s house/compound, tube well water, borehole, protected dug well, protected well, protected spring and rainwater collection, bottled water and sachet water. The unimproved source of drinking water in this study included unprotected wells, spring surfaces, unprotected springs, rivers/dams, tanker trucks and carts with small tanks. The type of toilet facility was also categorized into two categories: improved and unimproved. The improved toilet facilities in this study comprised flushing to pipe sewers, flushing to septic tanks, flushing to pit latrines, flushing to unknown places, flushing to biodigesters, ventilated improved pit latrines (VIPs), pit latrines with slabs, pit toilet latrines and composting toilets. The unimproved toilet facility included flush to somewhere else, pits without slab/open pit, no facility, bush/field and hanging toilet/latrines. The type of household cooking fuel was classified into the following: liquefied petroleum gas (LPG), charcoal, fuel wood and other cooking fuel (straw/shrub/grass, agricultural crops and animal dung).

### Statistical analysis

Data were analysed with Statistical Package for the Social Sciences (SPSS) version 25. In analysing the data, three stages were followed. The first stage was the use of simple descriptive statistics to describe the outcome variable, main predictor variable and other household factors. The second stage involved bivariate analyses sex of household head and other household factors associated with anaemia status in children aged 6–59 months using chi-square analysis. A *p* value of 0.05 was considered statistically significant. In the third stage, two models were developed that involved binary logistic regression analyses to examine the effect of sex of household head and other household factors on the anaemia status of children under the age of five. The first model examined the relationship between only sex of household head and childhood anaemia status while the second model examine the relationship between sex of household head and childhood anaemia status controlling for the effect of other household factors. The model included the adjusted odds ratios (aORs) and their associated 95% confidence intervals (CIs). The sample weight (v005/1,000,000) was applied in weighting all the data to correct possible over- and undersampling issues.

## Results

### Demographics characteristics and childhood anaemia status of households in Ghana

Table 1 shows the demographic characteristics and anaemia among children under age five of household factors for in Ghana. The results show that a relatively higher proportion of children under age five not anaemic (83.0%) compared to those who are anaemic (17.0%). Among those who are anaemic less than one percent were severely anaemia and about 8% had moderate and mild anaemia. More than half (65.2%) of households in Ghana are headed by males, and 23.0% (highest proportion) belonging to rich household wealth index. Majority of households in Ghana access improved source of drinking water (92.8%) and toilet facilities (74.7%). Most household heads are in the age category of 30–49 years and also uses fuel wood as the main source of cooking fuel. In Ghana, most household membership size is 1–4 members (65.4%) and more than half of households in Ghana own 1–3 insecticide-treated bednets. In terms of ecological zone of residence, almost half (49.5%) of households dwell in the middle belt zone of Ghana, while 51.5% resides in urban areas. Again only 7.1% of household reported that their dwelling had been sprayed against malaria in the past 12 months.

**Table 1:** Description of household characteristics and childhood anaemia in Ghana. *Source* Computed from the Ghana Malaria Indicator Survey (GMIS) of 2019

Childhood anaemia	Household data (weighted sample = 5,799)	
Anaemia status	Frequency	Percent
Not Anaemic	4,811	83.0
Anaemic	988	17.0
Anaemia levels		
Severe	18	0.3
Moderate	463	8.0
Mild	507	8.7
Household factors		
Sex of Household Head		
Male	3,782	65.2
Female	2,017	34.8
Wealth Index		
Poorest	861	14.9
Poorer	1,046	18.0
Middle	1,231	21.2
Richer	1,334	23.0
Richest	1,326	22.9
Household toilet facility type		
Improved	4,331	74.7
Unimproved	1,468	25.3
Household drinking water source		
Improved	5,381	92.8
Unimproved	418	7.2
Age of household head (years)		
20–29	1,070	18.5
30–39	1,422	24.5
40–49	1,181	20.4
50–59	947	16.3
60–69	642	10.8
70 +	555	9.6
Household cooking fuel		
Liquefied Petroleum Gas (LPG)	1,549	26.7
Charcoal	1,964	33.9
Fuelwood	2,005	34.6
Other cooking fuel	281	4.8
Number of household members		
1–4	3,795	65.4
5–10	1,897	32.7
11 +	106	1.8
Household bednets ownership		
No bednets	1,509	26.0
1–3 bednets	3,453	59.5
4 + bednets	837	14.4
Household Eco zone of residence		
Coastal/Southern zone	2,210	38.1
Middle Belt	2,869	49.5
Northern zone	720	12.4
Household place of residence		
Urban	2,984	51.5
Rural	2,815	48.5
House sprayed against malaria in the past 12 months		
Not sprayed	5,389	92.9
Sprayed	410	7.1

### Association between sex of household head, other household factors and childhood anaemia in Ghana

Table 2 illustrates a chi-square analysis between sex of household head, household factors and anaemia among children under age five in Ghana. Sex of household head was not significantly associated with childhood anaemia at *p* < 0.05. However, all other household factors considered in this study had a significant association with childhood anaemic status at *p* < 0.05.

**Table 2:** Association between sex of household head, other household factors and childhood anaemic status in Ghana. *Source* Computed from the Ghana Malaria Indicator Survey (GMIS) of 2019

Household factors	Childhood anaemia status		
	Not anaemic	Anaemic	*P* values
*Sex of Household head*			
Male	82.6	17.4	0.354
Female	83.6	16.4	
*Wealth Index*			
Poorest	69.7	30.3	< 0.001
Poorer	77.8	22.2	
Middle	81.9	18.1	
Richer	87.9	12.1	
Richest	91.7	8.3	
*Type of toilet facility*			
Improved	85.5	14.5	< 0.001
Unimproved	75.5	24.5	
*Source of drinking water*			
Improved	83.8	16.2	< 0.001
Unimproved	72.2	27.8	
*Age of Household Head*			
20–29	85.1	14.9	< 0.001
30–39	78.3	21.7	
40–49	80.3	19.7	
50–59	84.9	15.1	
60–69	87.5	12.5	
70 +	87.9	12.1	
*Type of cooking fuel*			
Liquefied Petroleum Gas	91.9	8.1	< 0.001
Charcoal	83.0	17.0	
Fuel wood	74.2	25.8	
Other fuel	95.7	4.3	
*Household membership size*			
1–4	91.1	8.9	< 0.001
5–10	68.2	31.8	
11 +	56.6	43.4	
*Bednets ownership*			
No bednets	91.5	8.5	< 0.001
1–3 bednets	82.0	18.0	
4 + bednets	71.7	28.3	
*Eco zone of residence*			
Coastal/Southern zone	86.2	13.8	< 0.001
Middle belt	84.7	15.3	
Northern zone	66.1	33.9	
*Place of residence*			
Urban	87.1	12.9	< 0.001
Rural	78.6	21.4	
*House sprayed against malaria in the past 12 months*			
Not sprayed	83.7	16.3	< 0.001
Sprayed	73.2	26.8	

There was a significant difference [*p* < 0.001] in childhood anaemia according to the household wealth index in Ghana. The results show that a high proportion of anaemic children in the poorest (30.3%) compared to those in the richest (8.3%) household wealth category. Similarly, high anaemia was recorded among children using unimproved toilet facilities (24.5%) and unimproved drinking water source (27.8%) compared to those accessing the improved ones.

The study also found that there are more anaemic children (21.7%) living with relatively young (30–39 years) heads compared to children (12.1%) living with relatively older heads (70 or more years).

In households where fuelwood is used for cooking, children recorded high anaemia (25.8%) compared to children in households using other types of cooking fuel. However, more anaemic children were found in household with 11 or more members (43.4%) compared to children in households with 1–4 membership (8.9%). With the ownership of household bednets, 28.3% of children in household owning four or more treated bednets were anaemic compared to 8.5% children who lived in households without treated bednets at *p* < 0.001. Children residing in households found in the northern zone (33.9%) recorded more anaemia than those living in the coastal/southern (13.8%) zone. A higher anaemia level was found among children residing in rural areas (21.4%) compared to urban children (12.9%). Interestingly, households who had their dwelling sprayed against malaria in the past 12 months had higher proportion of children being anaemic (26.8%) compared to those whose dwelling were not sprayed (16.3%).

### Sex of household head and other household factors predicting childhood anaemia status among households in Ghana

Table 3 shows the results of the sex of household heads and other household factors that predict the anaemic status among children aged 6–59 months dwelling in households in Ghana. In the first model, sex of household head did not significantly predict child anaemic status. However, in the second model, sex of household head significantly predicted the anaemic status of children under age five living in Ghana, after controlling for other household factors. Thus, household factors such as wealth index, age of the household head, type of cooking fuel, household membership size, ownership of bednets treated and ecological zone of residence of the household significantly predicted anaemia status of children under age five in Ghana.

**Table 3:** Odds ratios and confidence intervals of sex of household head and other household factors predicting anaemia status of children aged 6–59 months in in Ghana. *Source* Computed from the Ghana Malaria Indicator Survey (GMIS) of 2019

	Childhood anaemia status	
Variables	Model I aOR [95% CI]	Model II aOR [95% CI]
Household factors		
**Sex of Household head**		
Male	1.07 [0.93–1.24]	1.28* [1.08–1.51]
Female	Ref	Ref
**Household Wealth Index**		
Poorest		2.41*** [1.59–3.65]
Poorer		2.04*** [1.41–2.94]
Middle		1.78*** [1.29–2.46]
Richer		1.28 [0.96–1.72]
Richest		Ref
**Toilet facility type**		
Improved		1.01 [0.84–1.22]
Unimproved		Ref
** Source of drinking water**		
Improved		0.87 [066–1.13]
Unimproved		Ref
**Age of Household Head (years)**		
20–29		Ref
30–39		0.96 [0.76–1.22]
40–49		0.56*** [0.44–0.73]
50–59		0.39*** [0.30–0.52]
60–69		0.30*** [0.22–0.42]
70 +		0.31*** [0.22–0.43]
**Type of cooking fuel**		
Liquefied Petroleum Gas (LPG)		Ref
Charcoal		1.51** [1.15–1.99]
Fuel wood		1.44* [1.02–2.02]
Other cooking fuel		0.49** [0.21–0.78]
**Household size**		
1–4		Ref
5–10		4.49*** [3.78–5.34]
11 +		7.21*** [4.60–11.31]
** Bednets ownership**		
No bednets		0.57*** [0.44–0.74]
1–3 bednets		0.95 [0.78–1.16]
4 + bednets		Ref
**Eco zone of residence**		
Coastal/Southern zone		Ref
Middle Belt		0.93 [0.78–1.11]
Northern zone		1.40* [1.07–1.83]
**Place of residence**		
Urban		1.04 [0. 86–1.25]
Rural		Ref
**House sprayed against malaria in the past 12 months**		
Not sprayed		Ref
Sprayed		0.99 [0.74–1.33]

^*^P < 0.05, **P < 0.01, ***P < 0.001

Children who dwell in MHH had higher odds (aOR = 1.28; CI: 1.08–1.51) to be anaemic compared to those living in FHH after controlling for other household factors in the model.

The probability of a child being anaemic is high in the poorest (aOR = 2.41; CI: 1.59–3.65), poorer (aOR = 2.04; CI: 1.41–2.94) and middle (aOR = 1.78; CI: 1.29–2.46) in terms of wealth index compared to children in the richest households.

Regarding the age of household heads, the likelihood of being anaemic were lower among children whose household heads were 40–49 years (aOR = 0.56; CI: 0.44–0.73), 50–59 years (aOR = 0.39; CI: 0.30–0.52), 60–69 years (aOR = 0.30; CI: 0.22–0.42) and 70 years and above (aOR = 0.31; CI: 0.22–0.43) than children dwelling with heads aged 20–29 years.

Children in households using charcoal (aOR = 1.51; CI: 1.15–1.99) and fuelwood (aOR = 1.44; CI: 1.02–2.02) as cooking fuel are more likely to be anaemic than children in households that uses LPG. However, children dwelling in household that uses other types of cooking fuel had lower odds (aOR = 0.49; CI: 0.21–0.78) of being anaemic compared to those using LPG. Household with 5–10 members (aOR = 4.49; CI: 3.78–5.34) and 11 or more members (aOR = 7.21; CI: 4.60–11.31) will have their children more likely to be anaemic compared to children in the household of 1–4 members. Children in household without ownership of treated bednets (aOR = 0.57; CI: 0.44–0.74) had lower odds of being anaemic compared to children in households who owned 4 or more treated bednets. Higher odds of being anaemic were recorded among children residing in household located in the northern zone (aOR = 1.40; C.I:1.07–1.83) compared to children living coastal/southern households.

## Discussion

The study examined the effect of sex of household head and other household factors predicting anaemia status among children aged 6–59 months in households in Ghana using the 2019 GMIS data. The regression model indicates that children in MHH are more likely to be anaemic compared to children in FHH after controlling for other household factors. The high prevalence among children with MHH compared to FHH found in this study is contrary to the results from previous studies [25, 30, 31]. The explanations given by these studies for higher anaemia prevalence among children living in FHH are that when females become the household head in the absence of the husband in the household, all responsibilities in and outside the house are on her. In this situation, these women turn to focus more work outside the home and often allocate less time to take care of their children. Consequently, these children become malnourished and anaemic and are exposed to various forms of disease. In the context of this study, attention needs to be given to children in MHH, as the study found a high prevalence of anaemia among children from these households.

The study further found a high risk of anaemia among children living in the poorest, poorer and middle households compared to the richest household wealth index. The findings of this study are consistent with the findings of previous studies in Ghana [32, 33], sub-Saharan African countries [30], Malawi [16], Guinea [34] and India [35]. The likely explanation for this phenomenon is the assumption that poorer household heads in economically disadvantaged countries are economically stressed and nutritionally vulnerable to a greater extent than richer households. Again, it is generally asserted that rich household heads manage their resources differently because it is argued that they attach much importance to basic needs such as food and health care than poor household heads, and hence, richer household heads have the opportunity to enforce these priorities in the allocation of household resources.

Children who grew up with relatively older household heads were less likely to be anaemic compared to children with younger heads of household. Previous studies found similar results [36–38]. The possible explanation for this is that older heads have experience with childcare and are likely to take better care of children to prevent illness than younger household heads [37].

The use of hygienic fuel for cooking can also decrease the chances of anaemia among children [30, 35, 39]. While the type of cooking fuel used in MHH had a significant influence on childhood anaemia, there was no statistically significant relationship between the cooking fuel used in FHH and anaemia among children. In MHH, the likelihood of anaemia is higher among children whose household heads uses charcoal, and fuel wood compared to those using LPG. A reasonable explanation is that women often carry their children when cooking, and biofuel smoke carries high amounts of carbon monoxides, which transit oxygen to the tissues in the body, forming carboxyhaemoglobin and decreasing the ability of haemoglobin to carry oxygen, leading to anaemia [35].

The result further shows that the higher the size of household members, the more likely a child will become anaemic. Previous studies reported similar findings [40–43]. These studies found that a household with more than five persons is associated with a high risk of anaemia among children living in those households. The reasons were that households with relatively larger membership sizes tend to lack rich nutritional and dietary resources, which may affect the anaemia levels among children in these households.

Household heads with no ownership of insecticide-treated bednets were less likely to have their children being anaemic compared to heads who owned four or more bednets. This interesting finding is in line with the findings of other studies [26]. However, previous studies [44] found a high risk of childhood anaemia in a household with no bednets. Other studies did not find a significant association between household bednet ownership and the risk of childhood anaemia regardless of sex of the head [43]. Again, household heads who reside in the northern part of Ghana are more likely to have their children being anaemic compared to heads who are living in the southern/coastal areas.

### Limitations of the study

This study has some limitations. First, the GMIS is self-reported data, which may be subject to social desirability and recall biases. Additionally, there is the possibility of insufficient power to detect a statistically significant difference between MHHs and FHHs in the analysis. Finally, since this is a cross-sectional study, we are unable to establish causality between household factors and childhood anaemia in MHH and FHH in Ghana.

## Conclusion

The GMIS data suggest that anaemia and its severity are higher among children living in MHH than among those living in FHH. The results indicate that poverty, a higher number of household members, relatively younger male household heads and the type of cooking fuel used were factors accounting for the differences in anaemia severity between MHH and FHH. The findings of this study suggest that children living in MHH are more at risk of anaemia than children in FHH. Even though the literature on sex of household headship suggests that FHH is poorer and economically disadvantaged than MHH in Africa [45, 46], this study found otherwise. Equal attention should be given to MHH and FHH in terms of programmes and interventions aimed at preventing and reducing childhood anaemia in Ghana.

## Data Availability

Datasets used for this study are openly available and can be accessed through https://dhsprogram.com/

## Abbreviations

aOR: Adjusted odds ratio
CI: Confidence interval
DHS: Demographic and Health Survey
FHH: Female-headed household
GMIS: Ghana Malaria Indicator Survey
GPS: Global positioning system
GSS: Ghana Statistical Service
iCCM: Integrated community case management
ICF: Inner City Fund
IRB: Institutional Review Board
JMP: Joint Monitoring Programme
LPG: Liquefied petroleum gas
MHH: Male-headed household
MOH: Ministry of Health
NMCP: National Malaria Control Programme
NHIS: National Health Insurance Scheme
UNICEF: United Nations International Children’s Emergency Fund
WHO: World Health Organization
